# Nurses’ compliance with prevention of mother-to-child transmission national guidelines in selected sites in Kinshasa, Democratic Republic of Congo

**DOI:** 10.4102/phcfm.v7i1.844

**Published:** 2015-08-13

**Authors:** Augustin R.M. Amboko, Petra Brysiewicz

**Affiliations:** 1 School of Nursing and Public Health, University of KwaZulu-Natal, South Africa

## Abstract

**Background:**

The Democratic Republic of Congo (DRC) implemented a prevention of mother-to-child transmission (PMTCT) of HIV infection programme in maternal, newborn and child health (MNCH) services in 2001 with nurses as key personnel. To date there is no information in the DRC and specifically in Kinshasa with respect to compliance with PMTCT national guidelines.

**Aim:**

The study aimed at describing nurses’ compliance with the PMTCT national guidelines in selected PMTCT sites of Kinshasa.

**Methods:**

A descriptive cross-sectional study was conducted in Kinshasa with 76 nurses in 18 selected PMTCT sites. The nurses’ compliance with PMTCT national guidelines was assessed using a healthcare provider self-reporting questionnaire developed by the researchers.

**Results:**

The study showed that the mean score of nurses’ compliance with PMTCT national guidelines was 74% (95% CI: 69% – 78%) which progressively decreased and was significantly different across different MNCH services (*p* = 0.025). With respect to categories of PMTCT recommendations, nurses were compliant with those related to education in labour and delivery, and antenatal services. Sociodemographic characteristics such as training, length of service and category of nurses did not influence nurses’ compliance score.

**Conclusion:**

These findings showed that nurses were noncompliant with PMTCT national guidelines, with the score level being 80% or more in the three MNCH services/units. Improvement of nurses’ ‘compliance with the PMTCT national guidelines requires effective monitoring of full integration of PMTCT as routine activities in MNCH care.

## Abstract (fr)

**Respect des lignes directrices nationales de la prévention de la transmission mère-enfant (PTME) du VIH par les infirmères dans une sélection de sites de PTME à Kinshasa en RDC.**

**Contexte:**

La République démocratique du Congo (RDC) a mis en œuvre un programme de PTME dans des services de santé de la mère, du nouveau-né et de l’enfant en 2001 en considérant les infirmières comme les membres clés du personnel. À ce jour, il n’existe pas d’informations relatives au respect des lignes directrices nationales de PTME en RDC, et plus particulièrement à Kinshasa.

**But:**

L’étude visait à décrire le respect des directives nationales de PTME par les infirmières dans les sites de PTME sélectionnés de Kinshasa.

**Méthodes:**

Une étude transversale descriptive a été réalisée à Kinshasa auprès de 76 infirmières dans 18 sites de PTME sélectionnés. Le respect des directives nationales de PTME par les infirmières a été évalué à l’aide d’un questionnaire d’autodéclaration des fournisseurs de soins de santé élaboré par les chercheurs.

**Résultats:**

L’étude a montré que le score moyen associé au respect des directives nationales de PTME par les infirmières était de 74% (IC à 95 % : 69% − 78%), qui a progressivement diminué et était significativement différent entre les différents services de SMNE; *p* = 0.025). En termes de recommandations quant aux catégories de PTME, les infirmières étaient conformes à celles liées à l’enseignement relatif au travail, à l’accouchement et aux services de soins prénatals. Les caractéristiques socio-démographiques telles que la formation, la durée de service et la catégorie d’infirmières n’ont pas eu d’influence sur le score du respect des directives nationales de PTME par les infirmières.

**Conclusion:**

Ces résultats ont montré que les infirmières ne respectaient pas les directives nationales de PTME, le niveau de score étant de 80% ou plus dans les trois services/unités de SMNE. L’amélioration du respect des directives nationales de PTME par les infirmières nécessite un suivi efficace de la pleine intégration de la PTME en tant qu’activités de routine en soins de santé de la mère, du nouveau-né et de l’enfant.

## Introduction

In sub-Saharan Africa countries 230 000 children were newly infected in the perinatal period with HIV in 2013. Moreover, in 2012 210 000 children under 14 years of age died as a result of HIV infection.^1^ This situation is compromising the achievement of the fourth, fifth and sixth objectives of the Millennium United Nation's Development Goals in the African region.^2^ To address this challenge the World Health Organization recommended that each country implement a prevention of mother-to-child transmission (PMTCT) of HIV infection programme aimed at reducing the prevalence of HIV infection in children.^1,3,4^ The PMTCT programme was operationalised through the PMTCT guidelines, and the expected outcomes were to be achieved by complying with these.^5^

In most sub-Saharan African countries, however, there has been little progress in reducing mother-to-child transmission of HIV infection.^1,6^ In the Democratic Republic of Congo (DRC) a progressive increase of prevalence of HIV infection in newborns was reported, based on the UNAIDS spectrum estimate, from 23.2% in 2009^7^ to 23.3% in 2010^8^ and 38% in 2011,^9^ despite the programme being implemented in 2001 and having a high coverage, especially in Kinshasa. A prevalence of mother-to-child transmission (MTCT) of more than 20% reveals a lack of quality in implementation of PMTCT and its guidelines.^10^

There has been much research on PMTCT in most sub-Saharan African countries, including the DRC. However, this research focused on antenatal care (ANC) or postnatal care and did not review the entire continuum of maternal, newborn and child health (MNCH) in MNCH services.^11,12,13^ Whilst most studies addressed isolated PMTCT interventions, such as HIV testing, HIV testing and counselling, antiretroviral treatment (ART) and/or breastfeeding, they did not address comprehensive PMTCT interventions.^12,14,15,16,17^ Furthermore, the studies reviewed the records of the health services and targeted HIV-infected women, but gave little attention to the healthcare providers’ practices.^11,12,13,16,17,18,19^ More attention has been focused on monitoring PMTCT coverage, accessibility and utilisation rather than on standards of how PMTCT care is performed.^11,16,18,19,20,21,22^

One of major challenges that healthcare providers are experiencing is the gap between guidelines for prevention and treatment of HIV infection and compliance with these guidelines in clinical practice.^23^ It is therefore critical to investigate whether healthcare providers (especially nurses) are complying with the PMTCT national guidelines in MNCH services. With the DRC striving to move to MNCH services (which include ANC, labour and delivery, and postnatal/immunisation services) and a more effective PMTCT service, it is necessary to determine the baseline status of adherence to guidelines and identify areas of failure. This will also improve the quality of PMTCT care provided by nurses, who have become the main providers of care due to task shifting.

This article was part of a larger study undertaken by the researcher, which was aimed at describing the overall compliance with PMTCT national guidelines in selected PMTCT sites in Kinshasa, DRC.

## Research methods and design

### Study design

The study was conducted in Kinshasa from 15 December 2011 until April 2012 and employed a cross- sectional design.

### Setting

Kinshasa is the capital of the DRC. It is divided into 24 communes (municipalities), and in 2009 the population was estimated at 10 076 099.^24^ There are 154 PMTCT sites and approximately 2156 healthcare providers.^25,26^ At the time of the study only 35 PMTCT sites provided a continuum of PMTCT activities across the spectrum of ANC, labour and delivery to postnatal care and immunisation, where early detection of HIV infection by polymerase chain reaction (PCR) in infants six weeks after birth is done as part of the comprehensive PMTCT programme. The PMTCT sites are health facilities where MNCH care is offered, with trained staff of at least two healthcare providers per unit on integrated PMTCT/MNCH care, and a minimum package of activities (from HIV counselling and testing for pregnant women at ANC to dried blood spots [DBS] at 6 weeks after birth), and of material and equipment.

### Study population and sampling strategy

The researcher selected 50% of the 35 PMTCT sites providing the full PMTCT programme. The sample size was calculated using a conservative sample size estimate.^26^ As there was no prior knowledge with regard to the proportion of those who complied, the researcher assumed maximum possible variability (*p* = 0.5; *q* = 0.5 at 95% confidence interval and a precision (d) of ± 12%). The researcher then increased the sample size by 15% to take into account incomplete information,^26^ thus adjusting the sample size to 76 healthcare providers.

The stratified sampling method was used to select the sample of healthcare providers. In collaboration with the Provincial Programme to Control the HIV Epidemic and STIs, 18 PMTCT sites were randomly selected. Together with the PMTCT coordinator for Kinshasa, a list of all 35 sites was printed and each site name was put into a hat and then 18 sites were randomly selected. This selected sample had 278 healthcare providers, including 262 nurses, 7 doctors and 9 lay counsellors involved in PMTCT care at the selected PMTCT sites who worked in antenatal, labour and delivery and postnatal/immunisation services/units.

The sample population of 278 healthcare providers was divided into strata as the sample population for the stratum (PMTCT site) and then the proportion of the sample population for each stratum was determined. The sample size of each stratum was obtained by multiplying the proportion of each stratum with the sample size (76). The researchers randomly selected the overall sample of 76 healthcare providers in proportion to the size of the strata in the population, based on the unique printed list of all healthcare providers. Therefore all of the 76 healthcare providers who were selected were nurses.

### Data collection

The researchers developed a healthcare provider compliance questionnaire based on the DRC PMTCT national guidelines. These were categorised into patient education, treatment, diagnosis and follow-up/referral.^27^ The questionnaire consisted of 62 items, which were divided into three sections for the different areas of care. There were 26 items applicable to ANC, 19 items applicable to labour and delivery, and 17 items applicable to postnatal/immunisation care. The self-administered questionnaire used a five-point Likert scale. The instrument, developed in English, was translated into French and then back into English by a qualified translator.

The selected nurses were individually contacted by the researcher. Once they had provided written consent to participate in the study, each nurse was handed the section of the questionnaire that was relevant to their respective MNCH services. The nurses completed the questionnaires and returned them to the researcher on the same day.

### Data analysis

All data were captured and analysed using the Statistical Software Package for Social Sciences® (SPSS) version 19 (IBM Corp, Armonk, New York, 2010). Descriptive statistics were used to report the findings of the study and these included mean, median and interquartile range (IQR).

The healthcare providers’ compliance with the guidelines was defined as the percentage of PMTCT recommendations adhered to by healthcare providers at each level in the continuum of MNCH services according to the national guidelines as assessed by means of the self-reporting questionnaire. The study rated a score of 80% – 100% as indicating healthcare providers’ compliance with the relevant (to their place of work) section of the PMTCT national guidelines, and less than 80% as noncompliance. The researchers referred to Laurin's studies on quality of care^28^ and the UNAIDS international commitment^2^ to obtain parameters of delivering quality of care. Laurin set average quality at 70% – 84% and excellent quality of care at ≥ 85%, whilst the target of UNAIDS international commitment was at least 80% of HIV-infected women being provided with effective PMTCT services. Point estimate at 95% confidence interval has been performed for the nurses’ compliance scores.

Nonparametric statistics were performed. With nurse compliance as a dichotomous variable, a Chi-square test was used to test the association of nurses’ compliance with sociodemographic characteristic categories in the three services. Due to skewed data, Kruskal-Wallis tests were performed to assess differences of nurses’ compliance scores across the three services/units. A *p-*value of < 0.05 was deemed statistically significant.

### Validity and reliability

The instrument was tested for reliability and content validity. For reliability, the test-retest reliability coefficient (kappa) was more than 0.7,^29^ being 0.89, 0.78 and 0.72 for the antenatal, labour and delivery, and postnatal/immunisation questionnaires respectively.

For content validity, the questionnaire items fitted with the objectives of the study and the key concepts in the conceptual framework, including the experts’ opinion.^29,30^

### Ethical considerations

With the researcher being a student, ethical approval was sought and granted by the Humanities and Social Sciences Research Ethics Committee of the University of KwaZulu-Natal (reference: HSS/1265/011M). Approval and authorisation for conducting research in the DRC was granted by the Minister of Health of the Province of Kinshasa. Written informed consent in French was obtained from all respondents. Confidentiality was preserved throughout the study and data could not be traced back to individuals or sites.

## Results

The response rate was 100%. The sample realisation for healthcare providers was 76 nurses.

### Sociodemographic characteristics

Table 1 shows the sociodemographic characteristics of the nurses in the different MNCH services/units. The data revealed no significant differences across MNCH services/units. Most of the nurses were A2 nurses, with less than 10 years of experience (*p* = 0.522; *p* = 0.578), and were trained in the standardised PMTCT training package.

**TABLE 1:** Sociodemographic characteristics of nurses in different MNCH services/units.

Sociodemographic characteristics of nurses	Nurses per ward			*P-*value
	Antenatal ward *n* (%)	Labour and delivery ward *n* (%)	Postnatal ward *n* (%)	
**Categories of nurses**				
Nurses A2	15 (50%)	17 (61%)	8 (44%)	0.522
Nurses A1 and L2	15 (50%)	11 (39%)	10 (56%)	-
Length of service				
< 10 years	23 (77 %)	18 (64%)	13 (72%)	0.578
=/> 10 years	7 (23%)	10 (36%)	5 (28%)	-
**Training in PMTCT**				
Yes	24 (80%)	14 (50%)	9 (50%)	0.031
No	6 (20%)	14 (50%)	9 (50%)	-

MNCH, maternal, newborn and child health; PMTCT, prevention of mother-to-child transmission.

Nurse A2, nurse with diploma (4 years of secondary school + 4 years of nursing school); Nurse A1, nurse with bachelor degree (baccalaureate + 3 years); Nurse L2, nurse with bachelor and honours degree (baccalaureate + 5 years).

The median for years of working experience was 7 years in both ANC (IQR = 6) and postnatal/immunisation care (IQR = 15), but in labour and delivery care the median was 8 years (IQR = 9).

### Overall compliance of healthcare providers with prevention of mother-to-child transmission national guidelines

**FIGURE 1:**
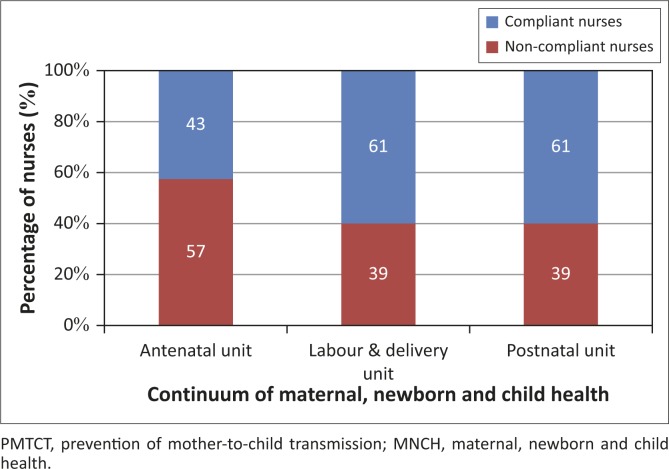
Proportion of nurses who were compliant and noncompliant with PMTCT national guidelines across the continuum of MNCH services. PMTCT, prevention of mother-to-child transmission; MNCH, maternal, newborn and child health.

Figure 1 presents the proportion of nurses who were either compliant or noncompliant with PMTCT national guidelines across the continuum of MNCH services. It shows that about 57% of nurses in the antenatal service complied with PMTCT national guidelines, as opposed to 39% in both the labour and delivery and postnatal services. In the bivariate analysis there was no significant association between nurses’ compliance scores with PMTCT national guidelines in the three MNCH services and their sociodemographic characteristics, such as professional category (*p* = 0.233), training in PMTCT (*p* = 0.821) and working experiences (*p* = 0.575).

Table 2 shows the nurses’ compliance with PMTCT national guidelines in the continuum of MNCH services. It revealed the mean score of nurses’ compliance with PMTCT national guidelines to be about 74% (95% CI: 69% – 78%).

**TABLE 2:** Mean score, median score and Kruskal-Wallis test scores of nurses’ compliance with PMTCT national guidelines on the continuum of MNCH services.

Categories of compliance score per ward	Mean (%)	95% CI [range (%)]	Median (%) IQR	*P*-value
Antenatal compliance with PMTCT national guidelines	80	[73–87]	85 [17]	0.025*
Labour and delivery compliance with PMTCT national guidelines	71	[64–78]	76 [27]	-
Postnatal compliance with PMTCT national guidelines	67	[56–79]	73 [34]	-
Overall compliance with PMTCT national guidelines	4	[69–78]	78 [22]	-

PMTCT, prevention of mother-to-child transmission; MNCH, maternal, newborn and child health.

*, *p* ≤ 0.05

The data aggregated by service indicate that nurses in ANC were compliant with antenatal PMTCT national guidelines, but that the mean score of nurses’ compliance with the section of the PMTCT national protocol relevant to the service where they worked progressively decreased from antenatal to postnatal service, respectively 80% (95% CI: 73% – 87%) in ANC; 71.3% (95% CI: 64% – 78%) in labour and delivery and 67% (95% CI: 56% – 79%) in postnatal/immunisation care. There is a significant difference in the scores of compliance in antenatal, labour and delivery, and postnatal/immunisation (continuum of MNCH) (*p* = 0.025).

### Compliance with PMTCT national guidelines by nurses and recommendations in the three MNCH services/units

The data with respect to PMTCT recommendations were skewed. Table 3 reflects the findings on nurses’ compliance with recommendations of PMTCT national guidelines in the three MNCH services.

**TABLE 3:** Mean and median scores and Kruskal-Wallis test of nurses’ compliance by categories of PMTCT recommendations and MNCH services.

Categories of recommendations of PMTCT national guidelines	Nurses per service	Mean (%)	95%CI [range, %]	Median (%) [IQR]	*P*-value
Compliance score (%) with education recommendations of PMTCT national guidelines	Antenatal service	85	[78–91]	91 [15]	0.000***
	Labour and delivery service	89	[79–99]	100 [0]	
	Postnatal service	72	[60–85]	83 [22]	
Compliance score (%) with treatment` recommendations of PMTCT national guidelines	Antenatal service	74	[62–86]	94 [50]	0.11
	Labour and delivery service	75	[67–82]	83 [25]	
	Postnatal service	63	[50–76]	67 [34]	
Compliance score (%) with diagnosis recommendations of PMTCT national guidelines	Antenatal service	64	[55–73]	66 [33]	0.29
	Labour and delivery service	72	[58–86]	94 [50]	
	Postnatal service	64	[44–83]	75 [59]	
Compliance score (%) with follow-up/referral recommendations of PMTCT national guidelines	Antenatal service	72	[55–88]	100 [100]	0.000***
	Labour and delivery service	-	-	-	
	Postnatal service	68	[47–88]	92 [75]	

PMTCT, prevention of mother-to-child transmission; MNCH, maternal, newborn and child health.

***, *p* ≤ 0.001

For PMTCT recommendations related to education, the mean score of nurses’ compliance was about 84% (95% CI: 78% – 91%) in ANC and 89% (95% CI: 79% – 99%) in labour and delivery. A very significant difference was found between the mean score of nurses’ compliance with PMTCT education recommendations across the continuum of MNCH (*p* = 0.000).

For the PMTCT recommendations related to treatment, diagnosis and follow-up/referral, the mean scores of nurses’ compliance were respectively 71% and 67% less than the threshold of 80% and more across the continuum of MNCH from antenatal to postnatal/immunisation services. Despite the noncompliance of nurses with PMTCT national guidelines, a significant difference was noted on compliance scores with follow-up/referral recommendations of PMTCT national guidelines across the MNCH services (*p* = 0.000).

Some sociodemographic characteristics also had a significant influence on nurses’ compliance with PMTCT recommendations. It was found that the nurses’ category was associated with the score of nurses’ compliance with PMTCT recommendations related to education (*p* = 0.034) and diagnosis (*p* = 0.043). However, no significant association was found with other PMTCT recommendations and sociodemographic characteristics.

## Discussion

The study found that the majority of nurses were trained in PMTCT and a significant proportion were noncompliant with the PMTCT national guidelines, which was much worse in the labour and postnatal care services compared to ANC. Furthermore, the treatment, diagnosis and follow-up/referral recommendations of PMTCT national guidelines on the continuum of MNCH, including the education recommendations in postnatal/immunisation services/units, constitute the weak domains of the PMTCT national guidelines in Kinshasa, DRC.

Noncompliance with healthcare standards is a common problem in developing countries,^31^ with studies in Malawi, Uganda, and India having similar findings.^10,17,21^ The exceptional nurses’ compliance in ANC may have been influenced by context, as when the PMTCT programme was first introduced in Kinshasa more focus was placed on ANC than delivery and postnatal care. A minimum package of improved ANC services in line with PMTCT was implemented in many of the health clinics in 2003, which included staff training and supervision.^11,32^ Thus the initial focus on ANC may have resulted in improved antenatal compliance.

The progressive decrease of compliance amongst nurses may express the failure in PMTCT in the DRC and shows that although the nurses receive pregnant women for delivery and mother-infant pairs in the postnatal and immunisation MNCH services, the PMTCT national programme has been poorly integrated in service support into the continuum of MNCH services.

Regarding the categories of PMTCT recommendations in the continuum of MNCH, the findings indicated that nurses were only compliant with the education recommendations in ANC and labour and delivery care, for example, providing individual post-test counselling after HIV testing (93.3%), providing information to HIV-positive women related to signs of labour and danger signs and MTCT timing (88.3%), and informing HIV-positive women regarding complete and regular immunisation according to the expanded programme on immunisation schedule for the baby with respect to clinical exceptions (88.3%) in ANC. In labour and delivery, for example, recommending early starting and follow-up of immediate exclusive breastfeeding after delivery (89.3%).

Furthermore, although in general nurses complied with the education recommendations, there were significant differences across the continuum of MNCH services. Other studies confirmed variations in this compliance, with nurses being more compliant to certain recommendations related to education than others; for example, Frizelle, Salomon and Rau underlined the noncompliance regarding breastfeeding information.^19,33^ This study found that nurses providing postnatal/immunisation PMTCT care were not complying with the education recommendations. Similar results were found in studies by Mazia et al.^13^ and Frizelle and Salomon^34^ in Botswana, Kenya, Malawi and Uganda. The researcher agreed with these authors, who pointed out that healthcare workers in their studies were provided with deficient messages related to PMTCT. There was poor quality of counselling and information was either contradictory, with avoidance of the topic or confusing messages told to mothers by healthcare providers.

Regarding the diagnosis recommendations, nurses’ compliance may be influenced by the type of strategy employed. The opt-out HIV testing strategy is more likely to influence healthcare providers’ compliance to provide HIV testing than the opt-in.^17^ The researchers suspected that nurses facing two ongoing HIV testing guidelines to either opt in or opt out may become confused. However, a systematic review and meta-analysis showed compliance where the CD4 count was done on the same day as HIV testing.^12,17,21^ In the context of the DRC, the noncompliance may also have been influenced by the need to pay for the CD4 count, as most HIV-related interventions are free. The link between nurses’ awareness about the need for the test and availability of the test might also play an important role.

However, the level of compliance with recommendations related to diagnosis in postnatal care depends, according to Grilli and Lomas,^35^ on the complexity of intervention. The DNA/PCR necessitates complex activities and special supplies which are not usually available at the PMTCT sites in the DRC. In contrast, the study by Tejiokem et al.^36^ in Cameroon found a high level of compliance with such interventions. The difference in these studies may be related to the location of the DNA/PCR test machine, availability of supply, the site and the health staff's roles in handling blood and infant policy- implemented diagnosis. For example, there is only one DNA/PCR test machine for early infant diagnosis in Kinshasa, challenging the transportation of DBS samples from the health districts to the capital of each of the 11 provinces in DRC and then to Kinshasa.

With respect to the treatment recommendations, the various options (nevirapine, zidovudine (AZT) and triple therapy-based regimens) available in the DRC may make it confusing for nurses recommending treatment, which may have led to their noncompliance with the guidelines.^7^ Higher compliance was reported where there was only one option (Eastern Cape, South Africa).^32^ Also, the poor integration of PMTCT into MNCH services may explain this reduced level of compliance. This occurs especially in labour and delivery, where invasive obstetric practices (use of forceps, rupture of membranes and suction of newborn) indicate that nurses have not been updated on MTCT progress.^37^

The study also found noncompliance with treatment recommendations in postnatal care. The study by Mazia et al. also reported low compliance with ART for newborns and HIV-positive women^13^ due to the challenge of packaging of ART medication for infants. Moreover, three ongoing and overlapping guidelines based on nevirapine, AZT and triple therapy in the DRC might also influence the level of compliance of nurses with this national guideline recommendation.

Regarding the follow-up/referral recommendations, the poor integration of PMTCT in the MNCH services disadvantaged the nurses’ ability to comply with the guidelines.^1,22,13^ These include, for example, immunisation of HIV-exposed infants for diphtheria, tetanus and pertussis at 6 weeks after birth, and recommendation of exclusive breastfeeding or exclusive infant formula feeding; also DBS samples of exposed infants were not routinely required, and were poorly performed.

The findings also took into account the sociodemographic characteristics of nurses. Although the nurses’ qualification had an influence on the score of nurses’ compliance (only with education and diagnosis recommendations of PMTCT national guidelines), in general nurses’ qualification, years of experience and training on PMTCT did not influence their overall level of compliance in the respective MNCH services. This confirms what has been described in the literature about factors which improve performance. A study in Armenia, Bangladesh, Bolivia, and Nigeria found that the social interactions (either formal or informal) between nurses and other professionals and experience are the most common sources of practice knowledge for nurses.^38^ Thus, in the current study the homogeneity in working experience amongst nurses in the three MNCH services/units and their formal interaction in the workplace might have had an influence on their overall poor compliance with PMTCT national guidelines.

### Limitations

The researchers assumed sample size estimate sampling due to no prior knowledge on healthcare providers’ compliance with PMTCT national guidelines in the continuum of care, which may not have been adequate. In addition, there was a lack of representativeness of all categories of healthcare providers involved in PMTCT. Nonetheless, the random selection of healthcare providers minimised the sample bias.

### Recommendations

More emphasis needs to be placed on the importance of evidence-based PMTCT guidelines in the nursing curriculum. This includes PMTCT management, nurses’ self-evaluation in daily practice and the full integration of all PMTCT recommendations as routine activities in MNCH. Attention should be focused on training more nursing staff in labour, delivery and postnatal services.

The nurses’ self-reported noncompliance with the diagnosis, treatment and follow-up/referral recommendations of the PMTCT guidelines requires further understanding. It is therefore recommended that further research could look at why noncompliance occurs from the nurses’ perspectives to address the problem and improve nurses’ compliance and implementation in order to reduce HIV infection in infants through PMTCT.

## Conclusion

The results of this study found that nurses were noncompliant with PMTCT national guidelines throughout the continuum of MNCH in the 18 selected PMTCT sites in Kinshasa, particularly in the labour and postnatal phase of care. Additionally, there is failure to comply adequately with the PMTCT national guidelines in providing diagnosis, treatment and follow-up recommendations across the PMTCT cascade.

The nurses’ self-reported noncompliance with the diagnosis, treatment and follow-up/referral recommendations of the PMTCT guidelines requires further understanding. In addition, more emphasis needs to be placed on the importance of evidence-based PMTCT guidelines in the nursing curriculum.
